# Does Glass Size and Shape Influence Judgements of the Volume of Wine?

**DOI:** 10.1371/journal.pone.0144536

**Published:** 2015-12-23

**Authors:** Rachel Pechey, Angela S. Attwood, Dominique-Laurent Couturier, Marcus R. Munafò, Nicholas E. Scott-Samuel, Andy Woods, Theresa M. Marteau

**Affiliations:** 1 Behaviour and Health Research Unit, Institute of Public Health, University of Cambridge, Cambridge, United Kingdom; 2 MRC Integrative Epidemiology Unit (IEU), UK Centre for Tobacco and Alcohol Studies, School of Experimental Psychology, University of Bristol, Bristol, United Kindom; 3 School of Experimental Psychology, University of Bristol, Bristol, United Kingdom; 4 Crossmodal Research Laboratory, Department of Experimental Psychology, University of Oxford, Oxford, United Kingdom; Oregon Health and Science University, UNITED STATES

## Abstract

**Background:**

Judgements of volume may influence the rate of consumption of alcohol and, in turn, the amount consumed. The aim of the current study was to examine the impact of the size and shape of wine glasses on perceptions of wine volume.

**Methods:**

Online experiment: Participants (n = 360; recruited via Mechanical Turk) were asked to match the volume of wine in two wine glasses, specifically: 1. the Reference glass holding a fixed reference volume, and 2. the Comparison glass, for which the volume could be altered until participants perceived it matched the reference volume. One of three comparison glasses was shown in each trial: ‘wider’ (20% wider but same capacity); ‘larger’ (same width but 25% greater capacity); or ‘wider-and-larger’ (20% wider and 25% greater capacity). Reference volumes were 125ml, 175ml and 250ml, in a fully factorial within-subjects design: 3 (comparison glass) x 3 (reference volume). Non-zero differences between the volumes with which participants filled comparison glasses and the corresponding reference volumes were identified using sign-rank tests.

**Results:**

Participants under-filled the wider glass relative to the reference glass for larger reference volumes, and over-filled the larger glass relative to the reference glass for all reference volumes. Results for the wider-and-larger glass showed a mixed pattern across reference volume. For all comparison glasses, in trials with larger reference volumes participants tended to fill the comparison glass less, relative to trials with smaller reference volumes for the same comparison glass.

**Conclusions:**

These results are broadly consistent with people using the relative fullness of glasses to judge volume, and suggest both the shape and capacity of wine glasses may influence perceived volume. Perceptions that smaller glasses contain more than larger ones (despite containing the same volume), could slow drinking speed and overall consumption by serving standard portions in smaller glasses. This hypothesis awaits testing.

## Introduction

Alcohol consumption is ranked 5th amongst the 20 leading risk factors for burden of disease in the world [1]. In addition to price, availability and marketing [2–4], other cues may also influence drinking behaviour and encourage people to drink more than they would otherwise. One such possible set of influences is the shape and size of glasses in which alcohol is served.

Perception of volume may be influenced by shape, in particular, by height (or length, depending on which is the most salient dimension). This is known as the “elongation effect”, whereby shorter cylinders have smaller perceived volumes than taller (or longer) cylinders of equal volume [5, 6]. These effects extend to containers used for beverages, where height tends to be most salient. For example, beer bottles are estimated to hold more than beer cans of the same volume, which tend to be shorter [7], and people pour more into shorter, compared with taller glasses, but perceive that they have poured less [8]. This seems also true of wine glasses: people have been shown to pour more wine into a wider wine glass than into a narrower wine glass [9].

Perception of volume is also clearly influenced by size, but changes in size may not result in corresponding changes in perceived volume: as the volume of cylinders increases, the influence of the “elongation effect” reduces [10], and size changes appear smaller when they vary in all three dimensions (height, width, depth) rather than just one of these [11]. The effect of size is further complicated when looking at glass size, particularly wine glass size, as not all glasses are filled to capacity. The evidence is mixed as to how glass size affects serving alcoholic beverages, with some studies showing larger glass sizes lead to larger amounts of alcohol being poured (by both students and experienced bartenders) [12, 13], with another finding no difference between amounts poured into smaller and larger wine glasses [9].

It is unclear whether perceptual effects on people’s judgements of volume relate to the height or size of the glasses themselves, or to the height or size of the volumes of wine contained within glasses, or both. In the context of food studies, plate size has been shown to affect perceptions of food volume, suggesting the relative fullness of tableware may influence volume judgements [14, 15]. Indeed, judgements of volume within incompletely filled glasses may also be subject to perceptual biases relating to how well people judge proportions, which has not been explored in this context. In particular, studies (using simple geometric shapes) suggest that proportions less than 0.5 are overestimated while the reverse holds for proportions greater than 0.5 [16, 17].

Such perceptual biases in volume judgements may affect consumption, for example by changing the rate at which a drink is consumed. Judgements of when half a beverage has been consumed have been suggested to guide people’s rate of drinking for alcoholic but not non-alcoholic drinks, with a trend suggesting an association between greater misjudgements of the midpoint of a glass (as lower than the true midpoint) and faster rates of drinking [18]. It is not yet clear if these misjudgements of the midpoint relate to the elongation effect. In the one study investigating this, when the midpoint was less accurately judged (with a curved glass), both the glass and a given portion within this glass had greater height than the comparison (straight-sided) glass [18]. Direct evidence for the influence of glass shape or size on consumption is limited, with studies comparing wider vs. narrower glasses (tumblers) so far yielding inconsistent results and no studies disentangling the effects of glass size from portion size [6, 8].

In summary, glasses may lead to perceptual biases for both alcoholic and non-alcoholic drinks. In addition, there is some evidence to suggest that the shape and size of glasses may influence the speed at which people consume alcohol and in turn the volume consumed. Given these possible additional consequences of glass selection for alcoholic drinks, alongside the health consequences of alcohol consumption, the focus of the current study is to expand on the results of previous work exploring perceptions of beer in different glasses [18], by investigating another popular alcoholic beverage: wine. Therefore, as an initial step in examining the potential influence of glass shape and size, this study examined perceived volumes of wine in glasses that vary in shape (wider vs. narrower) and size (smaller vs. larger). Three differently shaped and sized glasses were compared to a reference glass: (a) ‘wider’ (but same capacity); (b) ‘larger’ (but same width); (c) ‘wider-and-larger’, considered over three different reference volumes (125ml, 175ml and 250ml). We compared the study results to a set of hypotheses, outlined below, to explore which perceptual effects, or combination of effects, seemed most likely to be influencing judgements of volume.

### Hypotheses

#### Hypothesis 1 (Elongation effect: Height of wine)

First, due to the elongation effect, perceptions of volume in wine glasses may be affected by the height of the wine within the glass, such that greater height of wine leads to perceptions of greater volume. In terms of perceived volume (given the same actual volume) between glasses we would expect: *Wider glass = Wider-and-larger glass < Reference glass = Larger glass*. That is, in this study participants would be expected to over-fill the wider and wider-and-larger glasses relative to the reference glass, and fill the larger glass to match the reference glass.

#### Hypothesis 2 (Relative fullness effect: Glass size)

Alternatively, or in addition, the relative fullness of a glass might influence volume perceptions, with fuller glasses being seen as holding a greater volume (given the same actual volume), i.e. *Larger glass = Wider-and-larger glass < Wider glass = Reference glass*. However, perceptions of volume in wine glasses may not be consistently affected by what we term here the “relative fullness effect”, as volumes in glasses (perceived to be) fuller than 50% tend to be underestimated, and to be underestimated more as the glasses grow fuller [16, 17]. The impact of this bias is likely to vary according to both glass size and reference volume, both of which we directly manipulated.

#### Hypothesis 3 (Relative fullness effect & Size effect: Perceived glass size)

There may be an additional “size effect”, whereby as glasses change in multiple dimensions, these changes in size appear smaller [11]. As such, comparisons between the wider-and-larger glass, where both height and width differ to the reference glass, may be less salient than those for the larger glass, where the capacity change from the reference glass results only from increased glass height. That is, the predicted pattern of perceived volumes in Hypothesis 2 may be modified to: *Larger glass < Wider-and-larger glass < Wider glass = Reference glass*.

#### Hypothesis 4 (Elongation effect: Glass height & Relative fullness effect: Perceived glass size)

A final potential influence is an alternative consequence of the elongation effect that might result from the height of the glass itself influencing perceptions of glass size, which would lead to a contrasting hypothesis to that of the elongation effect for height of wine within glasses: *Larger glass < Reference glass < Wider-and-larger glass < Wider glass*. In this case participants would be expected to under-fill the wider and wider-and-larger glasses relative to the reference glass, and over-fill the larger glass relative to the reference glass.

## Methods

### Sample

Participants were US adults recruited via Mechanical Turk (n = 360). This sample size was pre-determined to give a power of more than 0.8 to detect small differences (d = 0.2) between the extent to which participants filled different glasses at p = 0.0055 (i.e. following Bonferroni correction for multiple comparisons). The majority of participants were males (67%, n = 242), with a mean age of 32.9 (range: 18–68). Most were highly educated with 60% (n = 217) being college graduates, 30% (n = 108) having had some college education but not to graduation, and 10% (n = 35) being high school graduates or less educated.

### Design

The study used a 3 x 3 within-subjects (comparison glass x reference volume) design, whereby participants completed trials for all glass comparisons (comparison glasses being: (a) ‘wider’ (but same capacity); (b) ‘larger’ (but same width); (c) ‘wider-and-larger’), containing each reference volume (125ml, 175ml, 250ml). This study was approved by the University of Cambridge Psychology Research Ethics committee (reference: Pre.2014.90), with participants giving written consent.

#### Glass stimuli

Glass stimuli were developed from a set of photographs taken of a 330ml wine glass (the reference glass) filled with red wine in small (1–4ml) increments. These photographs were then scaled separately in the vertical and horizontal dimensions so as to produce sets of photographs of comparison glasses that were: (a) 20% wider but of the same capacity (‘wider’); (b) the same width but 25% larger capacity (‘larger’); (c) 20% wider and 25% larger capacity (‘wider-and-larger’), while keeping other aspects of glass design as consistent as possible (given glass height and width determined glass capacity, glass height varied between the different glasses). Only the section of the photographs depicting the bowl of the glass was rescaled, adjusting the width of the photograph by scaling by a factor of 1.2 when width needed to be manipulated, and then adjusting height as necessary to achieve the correct capacity (see S1 Text for details). These rescaled images were then trimmed to a standard size (removing excess background from the images) and realigned with the bottom half of the image, depicting the glass stem, which was kept consistent throughout.

**Fig 1:**
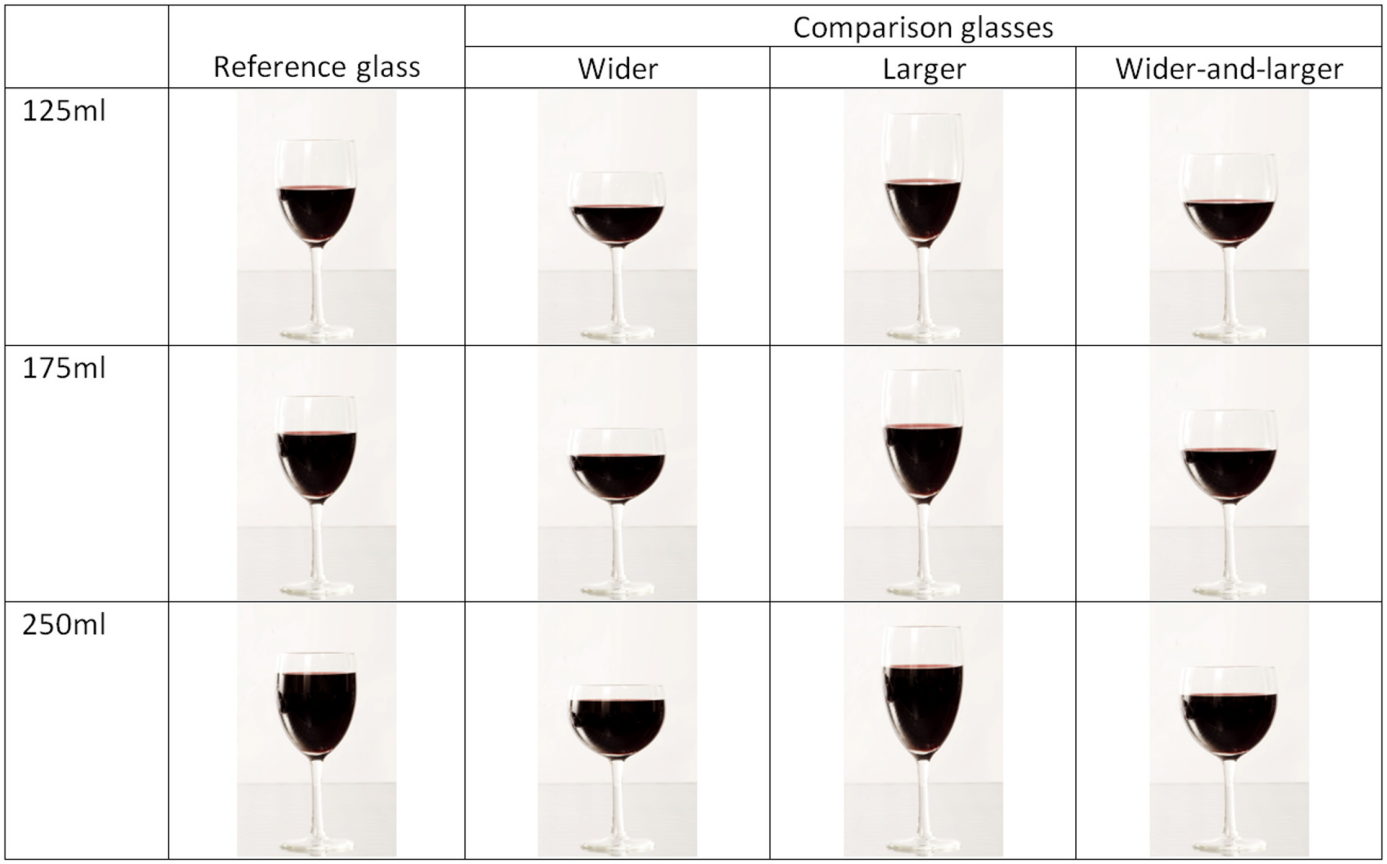
Reference volumes when presented in the different glasses.

Each of the sets of photographs was constructed so as to depict glasses filled in 5ml increments (resulting in a set of 67 photographs for the wider glass, and of 83 photographs for each of the other comparison glasses), i.e. photographs taken comprised the reference glass filled both to 5ml increments (for the reference glass images) and to volumes such that when rescaled the comparison glasses would be filled to 5ml increments. Fig 1 provides examples of the reference and comparison glasses containing each reference volume.

### Procedure

**Fig 2:**
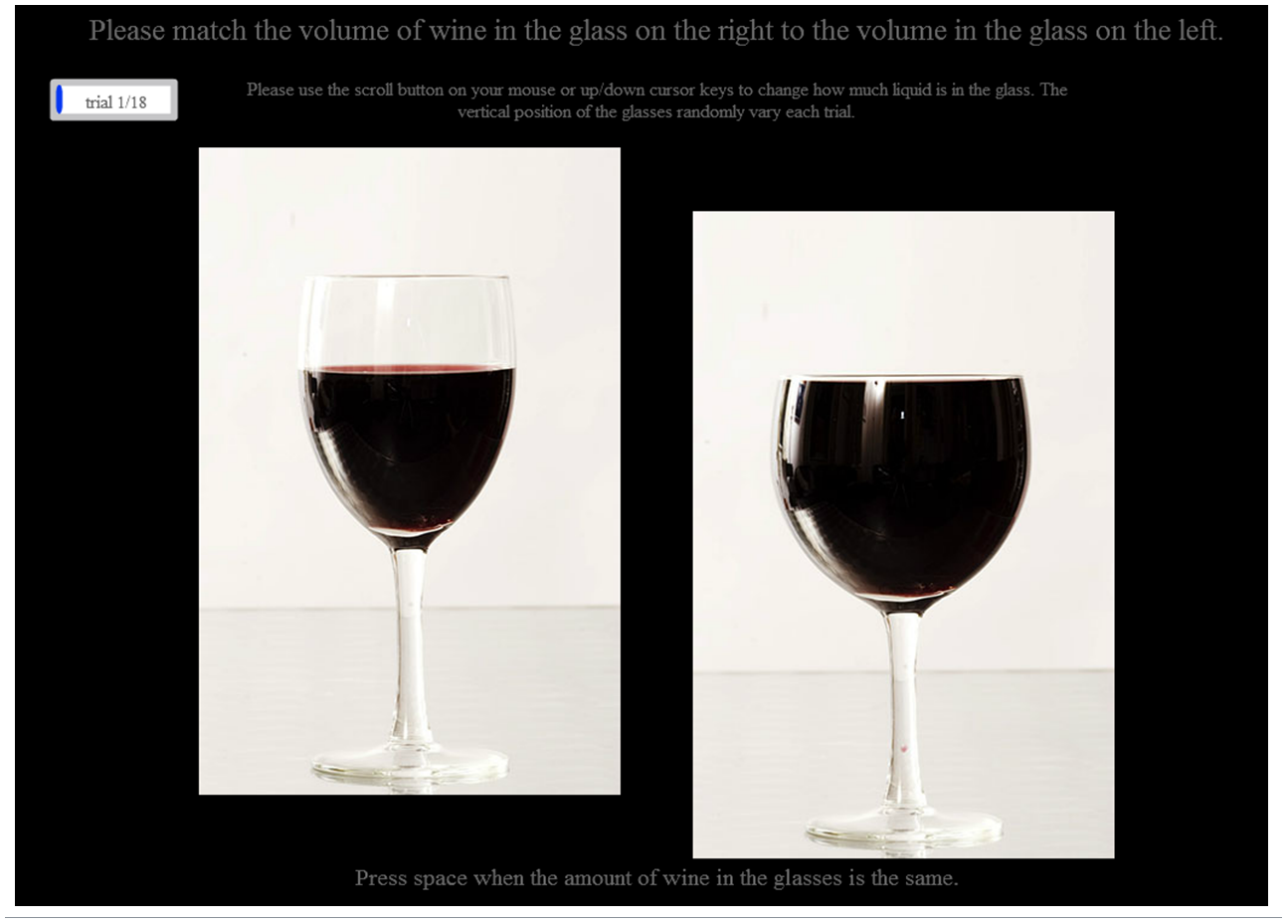
Example trial.

The study was completed online by participants using their own computers via the Xperiment platform (www.xperiment.mobi). After completing questions on basic demographic characteristics (age, gender and education), participants were presented with a series of trials, each comprising a picture of the reference glass containing one of the three reference volumes of interest, alongside a second picture of one of the three comparison glasses (see Fig 2). Participants were asked to adjust the volume of wine in the comparison glass so that it matched the volume in the reference glass, by scrolling with their mouse wheel. Scrolling changed the comparison glass image that was displayed, with volume changing by 5ml between each image, which appeared as a smooth filling or emptying of the glass depending on the direction of the scroll. Each participant completed 18 trials (3 comparison glasses x 3 reference volumes x 2: comparison glass full or empty at trial start).

Participants made judgements on two versions of each comparison glass by reference volume trial—one where they filled a comparison glass from empty, and one where they reduced the contents of the comparison glass from full—to avoid any hysteresis effects from participants adjusting volume from a single direction. Results from these ‘comparison empty’ and ‘comparison full’ trials were then averaged.

To prevent direct and easy comparisons of height of glasses and the volumes of wine displayed in glasses, trials included “vertical jitter” (whereby each of the two presented images was displayed misaligned from the vertical midpoint of the screen—one image above and one below—with each separately misaligned from the midpoint by a random factor of between 2–4% of the screen height). For each trial it was randomly determined whether the comparison glass was higher or lower than the reference glass due to the vertical jitter, and also whether the comparison wine glass was presented on the left or the right.

### Analysis

Differences between the volumes with which participants filled comparison glasses and the volume displayed in the reference glass were calculated for each trial. As would be expected as a result of known biases associated with hysteresis [19], trials in which the volume in the comparison glasses was adjusted from empty were consistently filled less than trials in which the volume in the glasses was adjusted from full (all p<0.05; Wilcoxon sign-rank tests). Rather than examine full and empty trials separately, which would give a biased estimate of the point of subjective equality, the average was taken for the trials in which the comparison glasses started empty or full, for each comparison glass x reference volume combination. Sign-rank tests were conducted to examine whether these averaged differences were different from zero (i.e. whether the amount of wine in the comparison glass that participants perceived to match the reference glass was different from the actual amount in the reference glass).

## Results

**Fig 3:**
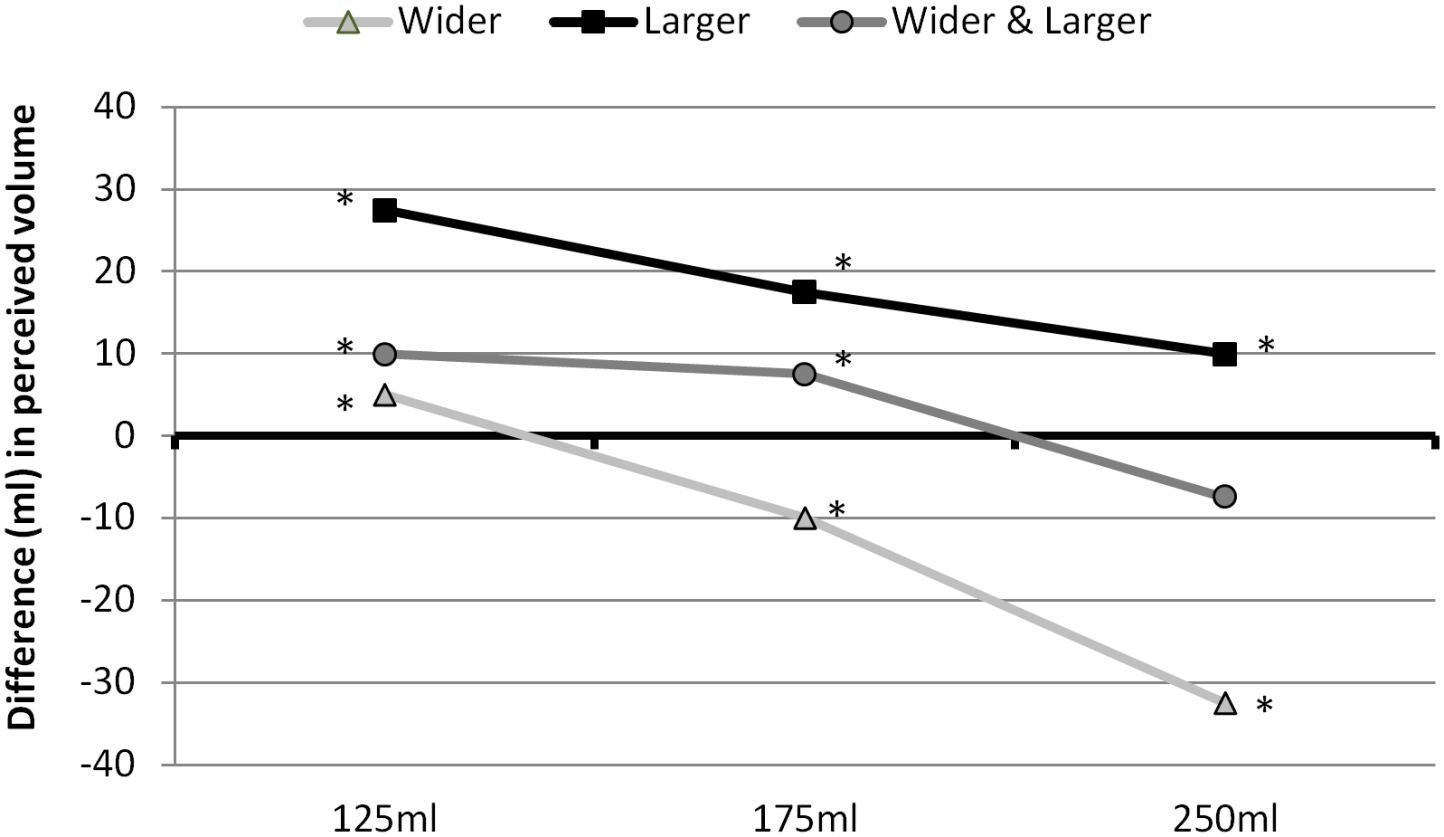
Median differences in volume perceived in ml (perceived matched volume in comparison glass minus actual volume in reference glass). Asterisks indicate significant differences between actual and perceived volumes (Bonferroni-corrected Wilcoxon sign-rank tests, testing difference from zero); all differences p<0.001.

The results of comparisons between the actual volume presented in the reference glass and the volume perceived to match this in the different comparison glasses are shown in Fig 3.

For the wider comparison glass, participants filled the volume in the reference glass more than necessary to match when it was filled to 125ml (average difference 5ml), but filled the wider glass less than necessary to match the reference glass for 175ml and 250ml reference volumes, with an average difference of 32.5ml for the 250ml reference volume compared to 10ml for the 175ml reference volume.

For the larger comparison glass, participants filled this glass more than necessary to match reference glass for all reference volumes, with the largest differences being seen for the smaller reference volumes (on average 27.5ml for the 125ml reference volume vs. 10ml for the 250ml reference volume).

For the wider-and-larger comparison glass, the pattern changed across reference volumes, with participants filling this glass more than necessary to match the reference glass for the smaller reference volumes (by 10ml on average for the 125ml reference volume and 7.5ml for the 175ml reference volume), and filling the comparison glass to match the 250ml reference volume.

## Discussion

Wider comparison glasses were filled less than necessary when matching larger reference volumes in the reference glass, suggesting these wider glasses were perceived to hold more wine given the same actual volume for these portion sizes. If the height of the wine in the glass was influencing judgements (due to the elongation effect: Hypothesis 1), wider comparison glasses would be perceived as containing less, given that the height of wine in these glasses is lower. An alternative explanation, more consistent with the observed results, is that the elongation effect instead leads participants to perceive the wider comparison glass itself as having a smaller capacity in general (despite actually having the same capacity), as a result of the smaller height of the glass (Hypothesis 4), which may affect perceived volume of the glass contents by making the glass appear fuller, with judgements in line with the relative fullness effect (Hypothesis 2). That is, rather than the glass shape affecting judgements of the fullness of the glass directly, it may affect perceived glass size in this instance.

As such, these results for glass shape may tie in with those seen in terms of glass size, where participants consistently perceived the larger comparison glass (which has greater capacity than the reference glass due to increased glass height) to contain less than the reference glass, in line with predictions made from the relative fullness effect (Hypothesis 2). However, results for the wider-and-larger comparison glass showed a mixed pattern across reference volume, suggesting some moderation of the relative fullness effect beyond that predicted from actual glass capacity. As with the wider comparison glass, this might reflect an effect of smaller glass height (than the reference glass) on perceived glass size, but for the wider-and-larger comparison glass this may be counteracted by the conflicting influence of greater actual glass capacity (Hypotheses 2 and 4). Alternatively, or in addition, this pattern of results for the wider-and-larger comparison glass might be incorporating some influence from the size effect (Hypothesis 3). Further work is needed to fully disentangle these effects, in particular for the wider-and-larger comparison glass.

The variation in perceived volume by reference volume further supports the hypothesis that relative fullness of a glass influences judgements of volume in wine glasses. In trials with larger reference volumes participants tended to fill the comparison glass less relative to trials with smaller reference volumes for the same comparison glass. This pattern of results by reference volume is largely consistent with previous findings on judgements of proportions, in which proportions greater than 0.5 tend to be underestimated, with this underestimation increasing until the proportion nears 1 [16, 17]. It is unclear, however, to what extent participants were accurately judging the midpoints of the glasses used in the current study. Indeed, the changing perceptions by reference volume are unlikely to solely reflect the influence of actual relative fullness of each glass on perceived volume, given that differences between the reference glass and the wider comparison glass also varied, despite the relative fullness of this comparison glass being identical to the reference glass for each reference volume. Instead, if participants perceive these glasses to have different capacities, they may also perceive the relative fullness of the glasses to be different even when these are actually the same.

These changes by reference volume are particularly important, as they suggest that the influence of glasses on drinking behaviour may vary by how full the glass is, and as such may not have a consistent effect on behaviour. Indeed, the pattern of results suggests that for some glass comparisons (e.g., wider), the glass that is perceived to contain more, given the same actual volume, may flip with changing reference volume. Investigating the influence of larger vs. smaller glasses on perceptions of smaller volumes may be particularly beneficial, to allow exploration of the consistency of effects as people face decreasing amounts in their glasses, akin to what individuals experience when drinking a glass of wine. That said, previous work on rates of consumption suggests that the speed at which the first half of an alcoholic drink is drunk may determine overall speed of consumption [18]. If so, it is the perceptual differences of glasses between their midpoint and full capacity that may have the greatest influence on wine drinking behaviour.

An additional consideration is the differences observed when trials began with a full vs. an empty comparison glass. It is possible such biases may come into play at different stages associated with drinking behaviour—for example, pouring into an empty glass may lead people to pour less to match a portion in a similar glass compared to someone drinking from a full glass, if trying to match the same portion in both scenarios. Nevertheless, given this empty vs. full bias is consistently found across comparison glass and portion size, this may have only limited effects on the general pattern of perceptual biases suggested in the study, although the smaller positive differences observed may not be apparent when adjusting volumes from empty, and similarly for smaller negative differences from full. As such, it is possible that this bias might lead to different pouring or drinking behaviour in particular circumstances, but the results of the current study are likely to reflect the average perceptual differences between the investigated glasses at each portion size.

The current study is the first to examine perceptions of the volume of wine in differently sized and shaped wine glasses, as well as the potential mechanisms that might be driving such effects. It demonstrates that the relative fullness of glasses appears to have a greater influence on perceptions than the height of wine in the glass, at least for this selection of glasses and reference volumes. Indeed, by including a range of reference volumes (representing the portion sizes commonly served in the UK), this study revealed the potential moderation of effects of glass size or shape depending on glass fullness, highlighting the need for greater nuance in discussions of perceptual biases in this context. Moreover, by systematically varying aspects of size and shape, this study was able to examine whether there were distinct effects of these different aspects of glass design that have often been confounded in previous studies of wine glasses.

Several limitations of this study should be noted. Firstly, the judgements are made on photographs rather than on actual glasses (given that the actual glasses that would have allowed us to disentangle the effects of size and shape do not exist), and it is possible that the two-dimensional nature of these images affected results. Evidence to suggest this may not be a problem comes from studies of food judgements. Judgements of portion sizes of different foods based on photographs shows general consistency of findings between studies and when comparing perceptions of portion size of physically present food to photographs [20–22]. Second, participants completed the study on their own computers or smart phones, which would have varied in terms of display quality and size, and may have impacted on participants’ ability to make judgements. Third, in terms of the generalisability of results, it is unknown whether the conclusions from the current study would carry over to other types of glasses or beverages. Given previous studies have suggested that perceptual effects on consumption may differ between alcoholic and non-alcoholic beverages [18], the perceptual effects themselves may also differ between alcoholic and non-alcoholic beverages. Future research could address the question of whether the current findings may be limited to perceptions of wine. Finally, the implications of these results for alcohol consumption are limited by a lack of evidence on how such perceptual biases influence actual pouring or drinking behaviour. If judgements of volume in a glass do change speed of drinking, reflecting previous findings linking greater misjudgements of glass midpoints and faster rates of drinking beer [18], they may influence wine consumption over a period of time (as yet unestablished). Any such effects may, however, be curbed if personal consumption norms override any influence of perceptual bias or rate of consumption [23]. This might occur if people have a “unit bias” (i.e. they routinely drink a pre-set number of glasses of wine regardless of the perceived size of these glasses) [24, 25].

Studies are needed to fully disentangle the different possible effects that might be contributing to differences in judgements observed in this study, assessing how they interact with changing volumes. Studies are also needed to examine whether, when and how judgements of volume influence wine consumption as a basis for interventions designed to reduce consumption across populations.

## Supporting Information

S1 Dataset Judgements of Volume of Wine Dataset. (CSV)

S1 Text Calculation of Glass Capacity. (DOCX)
