# Supplementary material for: Does Glass Size and Shape Influence Judgements of the Volume of Wine?
Source: PLoS One. 2015 Dec 23;10(12):e0144536. doi: 10.1371/journal.pone.0144536 (PMC4689536; doi:10.1371/journal.pone.0144536)
Supplement: S1 Text — (DOCX) [file pone.0144536.s002.docx]

**S1 Text. Calculation of Glass Capacity.**

Capacity was determined by assuming that the part of the glasses to be morphed is trapped in a cuboid and represents a given fraction of its volume. The volume of the morphed glasses, after modifying the width, height and depth dimensions of the cuboid, may be deduced by multiplying the fraction of the glass by the volume of the morphed cuboid. Let x_0_, y_0_ and z_0_ respectively denote the width, height and depth of the original cuboid, and let alpha denote the fraction of the volume of the wine in the cuboid. Then, the volume of wine in the original glass, v_0_, is given by: v_0_ = α x_0_ y_0_ z_0_; and the volume of wine in the morphed glass, v_m_, is given by: v_m_ = α β_x_x_0_ β_y_y_0_ β_z_z_0_ = β_x_ β_y_ β_z_ v_0_, where β_x_x_0_, β_y_y_0_ and β_z_z_0_ respectively denote the width, height and depth of the modified cuboid. As β_x_ = β_z_, we finally have that v_m_ = β_x_^2^ β_y_ v_0_.
